# Cardiorespiratory responses to muscle metaboreflex activation in fibrosing interstitial lung disease

**DOI:** 10.1113/EP090252

**Published:** 2022-03-30

**Authors:** Charlotte Chen, John Kolbe, Margaret L. Wilsher, Sally De Boer, Julian F. R. Paton, James P. Fisher

**Affiliations:** ^1^ Manaaki Manawa – The Centre for Heart Research Department of Physiology Faculty of Medical & Health Sciences University of Auckland, Auckland New Zealand; ^2^ Department of Medicine Faculty of Medical & Health Sciences University of Auckland Auckland New Zealand; ^3^ Respiratory Services Auckland District Health Board Auckland New Zealand

**Keywords:** dyspnoea, exercise tolerance, group III/IV afferents, interstitial lung disease

## Abstract

**New Findings:**

- **What is the central question of this study?**
  We determined whether sensory feedback from metabolically sensitive skeletal muscle afferents (metaboreflex) causes a greater ventilatory response and higher dyspnoea ratings in fibrosing interstitial lung disease (FILD).
- **What is the main finding and its importance?**
  Ventilatory responses and dyspnoea ratings during handgrip exercise and metaboreflex isolation were not different in FILD and control groups. Blood pressure and heart rate responses to handgrip were attenuated in FILD but not different to controls during metaboreflex isolation. These findings suggest that the muscle metaboreflex contribution to the respiratory response to exercise is not altered in FILD.

**Abstract:**

Exercise limitation and dyspnoea are hallmarks of fibrosing interstitial lung disease (FILD); however, the physiological mechanisms are poorly understood. In other respiratory diseases, there is evidence that an augmented muscle metaboreflex may be implicated. We hypothesized that metaboreflex activation in FILD would result in elevated ventilation and dyspnoea ratings compared to healthy controls, due to augmented muscle metaboreflex. Sixteen FILD patients (three women, 69±14 years; mean±SD) and 16 age‐matched controls (four women, 67±7 years) were recruited. In a randomized cross‐over design, participants completed two min of rhythmic handgrip followed by either (i) two min of post‐exercise circulatory occlusion (PECO trial) to isolate muscle metaboreflex activation, or (ii) rested for four min (Control trial). Minute ventilation (${\dot{V}}_{E}$; pneumotachometer), dyspnoea ratings (0–10 Borg scale), mean arterial pressure (MAP; finger photoplethysmography) and heart rate (HR; electrocardiogram) were measured. ${\dot{V}}_{E}$ was higher in the FILD group at baseline and exercise increased ${\dot{V}}_{E}$ similarly in both groups. ${\dot{V}}_{E}$ remained elevated during PECO, but there was no between‐group difference in the magnitude of this response (Δ${\dot{V}}_{E}$ FILD 4.2 ± 2.5 L·min^–1^ vs. controls 3.6 ± 2.4 L·min^–1^, *P* = 0.596). At the end of PECO, dyspnoea ratings in FILD were similar to controls (1.0 ± 1.3 units vs. 0.5 ± 1.1 units). Exercise increased MAP and HR (*P* < 0.05) in both groups; however, responses were lower in FILD. Collectively, these findings suggest that there is not an augmented effect of the muscle metaboreflex on breathing and dyspnoea in FILD, but haemodynamic responses to handgrip are reduced relative to controls.

## 1 INTRODUCTION

Interstitial lung diseases (ILDs) are a heterogeneous group of conditions which arise from inflammation and/or fibrosis of the lung parenchyma and have an estimated prevalence of 5—18 per 10,000 people (Broaddus et al., 2016). ILDs share similar features of pulmonary architectural distortion, fibrosis, lung volume restriction, and impaired gas exchange (Broaddus et al., 2016). Exercise limitation is a key symptom in ILD and can be associated with dyspnoea (Broaddus et al., 2016; Holland, 2010). This is important because exercise capacity has a powerful prognostic value in terms of survival (Caminati et al., 2009; Kawut et al., 2005; Lederer et al., 2006) and is associated with poor health‐related quality of life in idiopathic pulmonary fibrosis (IPF), the most extensively studied ILD (Olson et al., 2016; Verma et al., 2011). Unfortunately, few interventions have produced sustained improvements in exercise tolerance (Holland, 2010). Studies exploring potential novel therapeutic targets are required.

Exercise hyperpnoea is the fundamental ventilatory response to exercise. However, the control of breathing during exercise is multifactorial and incompletely understood (Dempsey et al., 2014). Although historically controversial, there is accumulating evidence supporting the role of group III and IV skeletal muscle afferent feedback in human exercise hyperpnea (Bruce et al., 2019; White & Bruce, 2020). Group III and IV muscle afferents are activated during exercise by mechanical and metabolic intramuscular stimuli, and afferent signals are transmitted to medullary cardiorespiratory centres via the spinal cord (Fisher et al., 2015; Kaufman & Forster, 1996). Augmented skeletal muscle afferent activation has been identified in several disease states (e.g., heart failure, hypertension) and can result in marked sympathetic vasoconstriction, metabolic distress and exercise intolerance (Vianna & Fisher, 2019). Furthermore, there is evidence of abnormal involvement of group III and IV skeletal muscle afferents in the control of ventilation in conditions characterized by exertional dyspnoea, such as heart failure and chronic obstructive pulmonary disease (COPD) (Bruce et al., 2016; Gagnon et al., 2012; Piepoli et al., 1996). Indeed, the isolated activation of metabolically sensitive afferents (metaboreflex) using post‐exercise circulatory occlusion (PECO), by the inflation of a cuff to a supra‐systolic pressure immediately prior to the end of exercise and throughout recovery thereby trapping exercise‐induced metabolites, produced a marked elevation in ventilation in heart failure (+169%) and COPD patients (+27%) (Bruce et al., 2016; Piepoli et al., 1996). Similarly, intrathecal fentanyl (μ‐opioid agonist), administered to selectively attenuate group III and IV skeletal muscle afferent feedback, alleviated exertional dyspnoea and enhanced exercise capacity in COPD (Gagnon et al., 2012). Despite evidence for the involvement of aberrant skeletal muscle afferent activation in the pathogenesis of exertional dyspnoea, it remains unknown whether skeletal muscle afferent sensitivity is altered in ILD.

Herein, we recruited patients with fibrosing ILD (FILD), a disease subtype characterised by increased risk of progressive decline in lung function and early mortality (Cottin et al., 2019). We aimed to determine the cardiorespiratory responses to isolated metaboreflex activation with PECO in FILD. Using a randomized crossover design, patients and controls underwent trials of rhythmic handgrip exercise followed by either PECO or free‐flow recovery. Handgrip exercise was used to avoid ventilatory mechanical constraints, which may result during dynamic exercise with a larger muscle mass in FILD (Faisal et al., 2016). We tested the hypothesis that metaboreflex activation results in elevated ventilation and ratings of dyspnoea in FILD compared to healthy controls. In addition, given the contribution of group III and IV skeletal muscle afferents to the cardiovascular and autonomic responses to exercise (Vianna & Fisher, 2019), heart rate and blood pressure responses to isolated metaboreflex activation, and their associations with indices of cardiac autonomic control, were assessed.

## 2 METHODS

### 2.1 Ethics approval

This study was approved by the Health and Disability Ethics Committee, New Zealand (20/NTA/68) and prospectively registered in the Australian New Zealand Clinical Trials Registry (ACTRN12620001018909). Written informed consent was obtained from all participants following verbal and written explanation of the study procedures. The study was conducted according to the Declaration of Helsinki.

### 2.2 Participants

Sixteen patients with stable (no exacerbations in the preceding 6 weeks) FILD were recruited from a specialist tertiary ILD clinic in Auckland, New Zealand. Eligibility criteria were physiologic evidence of restriction (total lung capacity of < 80% of predicted, forced expiratory volume in one second/forced vital capacity ratio of > 0.7) and high‐resolution computed tomography evidence of pulmonary fibrosis (Sverzellati *et al*., 2015). Subjects were excluded if they had more than 10 pack‐year smoking history, emphysema on chest computed tomography, pulmonary sarcoidosis or had regular inhaled bronchodilator treatment for airways disease. Other exclusion criteria were body mass index ≥ 35 kg/m^2^, significant comorbidities other than FILD that may contribute to dyspnoea and/or reduce exercise capacity, infectious illness, pregnancy, current users of recreational drugs and abusers of alcohol. Sixteen healthy age‐ and sex‐matched controls were also recruited. They were free from respiratory disease and any comorbidities that may contribute to dyspnoea and/or reduce exercise capacity. Other exclusion criteria were identical to the FILD participants.

All participants attended an initial familiarization visit to determine eligibility. Anthropometric and general health information was collected during this visit. Activity‐related dyspnoea was assessed using Modified Medical Research Council Dyspnoea Scale (Fletcher et al., 1959). Spirometry (Spirolab, Medical International Research, Rome, Italy) was performed according to guidelines (Miller et al., 2005). FILD participants’ most recent static lung volumes (by body plethysmography) and test of gas transfer were collected from electronic medical records. The Global Lung Function Initiative reference sets were used for percentage predicted values for spirometry and gas transfer (Hall et al., 2021; Quanjer et al., 2012; Stanojevic et al., 2017), and the European Community for Coal and Steel reference sets were used for body plethysmography lung volumes (Quanjer et al., 1993).

### 2.3 Experimental design

The study followed a randomized cross‐over design. At least 2 days following the familiarization visit, participants attended the laboratory for the experimental visit. Participants refrained from food consumption for 2 h, caffeine, alcohol, and exercise for 12 h prior to the visit. Participants were seated and held a handgrip dynamometer (ADInstruments, Bella Vista, NSW, Australia) with their right hand. The dynamometer was supported by a custom‐made frame placed on a bedside table. The participants’ right arm rested comfortably on the same table. Maximum voluntary contraction (MVC) was determined by instructing the participants to perform 3–5 maximal handgrip efforts (over one second) separated by ∼1 min. The highest was taken as the MVC.

Participants performed two trials during the experimental visit. The order was randomized, and trials were separated by a 30‐min recovery period. Prior to each trial, participants rested for 5 min to establish steady state physiological variables. Trials began with a 2 min resting baseline followed by 2 min of rhythmic handgrip exercise with the right hand consisting of a 1 s contraction at 50% MVC followed by a 1 s relaxation (Bruce et al., 2016). The timing of the contraction and relaxation was guided by a metronome. Participants matched their handgrip force to a target force displayed on a computer screen. Following handgrip exercise, participants either rested for 4 min (Control) or a cuff placed around the upper right arm was rapidly inflated to 200 mm Hg starting 2–3 hand grip contractions before the end of the exercise period (PECO). The cuff was deflated after 2 min, and participants then rested for a further 2 min. Rhythmic handgrip was chosen as the exercise modality due to the relatively limited exercise capacity of the study population. It permits reliable isolation of metaboreflex with PECO and has been successfully used COPD (Bruce et al., 2016).

### 2.4 Cardiorespiratory measures

Participants wore an oronasal mask (Hans Rudolph Inc., Shawnee, KS, USA) attached to a heated pneumotachograph (3830 Series, Heated Linear E Pneumotachometer; Hans Rudolph). Respiratory airflow was continuously measured, and from this, breath‐by‐breath respiratory frequency (R*f*), tidal volume (V_T_) and minute ventilation (${\dot{V}}_{E}$) were calculated. All volumes recorded were converted to BTPS. The end‐tidal partial pressure of CO_2_ (P_ET_CO_2_) was measured at the oronasal mask with a gas analyser (Respiratory Gas Analyzer, ML206, ADInstruments, Bella Vista, NSW, Australia). Heart rate (HR) was monitored using a 3‐lead electrocardiogram (BioAmp, FE231, ADInstruments). Blood pressure was measured beat‐to‐beat using finger photoplethysmography (Human NIBP Nano interface, MLA382, ADInstruments) with the cuff placed on the middle or index finger of the left hand. Finger blood pressure was validated with automated digital sphygmomanometer brachial artery blood pressure measurements (Tango M2 BP monitor, SunTech, Morrisville, USA). Peripheral oxygen saturation (SpO_2_) was monitored with finger pulse oximetry (MLT321 and ML320/F, ADInstruments). Due to technical reasons beat‐by‐beat blood pressure and SpO_2_ were unavailable for one FILD participant.

Dyspnoea ratings were assessed using Borg's 0 to 10 category ratio scale (Borg, 1982), with 0 corresponding to ‘no difficulty at all with breathing’ and 10 corresponding to ‘maximal difficulty with breathing’. Participants were asked to provide ratings at rest. Then at the end of each trial, participants were asked to recall the rating at the end of the handgrip exercise period, at the end of the cuff occlusion period (PECO trial) and in the middle of the recovery period (Control trial). Participants were also asked to recall their rating of perceived exertion (RPE) at the end of the handgrip period, using modified Borg RPE scale, with 0 corresponding to ‘no perceived exertional at all’ and 10 corresponding to ‘maximal perceived exertion’.

### 2.5 Data analysis

Data were acquired at 1 kHz using a Powerlab 16/35 data acquisition system and LabChart Pro software (ADInstruments). Mean values for ${\dot{V}}_{E}$, P_ET_CO_2_, blood pressure, HR and SpO_2_ during each minute of both trials were calculated. The change in key cardiorespiratory values from baseline during each minute were also determined. Pulse pressure was calculated as systolic minus diastolic blood pressure. Mean arterial pressure (MAP) was calculated as one third of pulse pressure plus diastolic blood pressure.

An index of the muscle metaboreflex response was determined as the average intra‐individual difference in physiological response between the last 90 s of the PECO period and the corresponding period of the Control trial (Piepoli et al., 1996). For example, the metaboreflex contribution to ${\dot{V}}_{E}$ in FILD group is the mean of each FILD participant's Δ${\dot{V}}_{E}$ PECO period ‐ Δ${\dot{V}}_{E}$ control period.

In order to provide an indication of resting cardiac autonomic control, spontaneous cardiac baroreflex sensitivity (cBRS) and heart rate variability (HRV) were evaluated using data from the baseline of both trials. Only participants in sinus rhythm were included in the analysis (*n* = 3 FILD participants excluded). cBRS was calculated using the sequence technique (Parati *et al*., 2000) with CardioSeries software (v2.7, Ribeirão Preto, SP, Brazil). HRV was evaluated using both time‐domain and frequency‐domain analysis (Kubios HRV Standard, v 3.5.0, Kubios Oy, Kuopio, Finland). Time domain measures were square root of the mean of the sum of successive differences in R‐R interval (RMSSD) and standard deviation of all normal sinus R‐R internals (SDNN). Fast Fourier transformation of R‐R variability was used for the frequency‐domain analysis, and the power spectra were quantified by the following: very low frequency power (0.0–0.04 Hz), low‐frequency power (LF; 0.04–0.15 Hz), and high‐frequency power (HF; 0.15–0.4 Hz) (Anonymous, 1996). Cardiac vagal activity is a major contributor to the HF component whilst the LF component consists of both sympathetic and parasympathetic influences (Anonymous, 1996).

### 2.6 Statistical analysis

The comparison of participant group characteristics was undertaken using an independent sample t‐test. Two‐way analysis of variance (ANOVA) was used to examine baseline cardiorespiratory data for the Control and PECO trials. For time series data, the main effects of group, time and their interaction were examined using two‐way ANOVA with repeated measures. Normality of residuals were verified by visualisation. Post hoc analysis, when appropriate, was carried out using a Students t‐test with a Bonferroni correction. Comparisons between the control and FILD groups for muscle metaboreflex response were assessed using an independent sample t‐test. Pearson's correlation coefficient was used to determine whether a relationship existed between metaboreflex response and FILD severity. For cBRS and HRV analyses, comparison between groups was assessed using a t‐test. Natural log transformation was applied for skewed distributions. Non‐normally distributed data were assessed using Mann Whitney U test. Correlation between resting HRV and HR responses to handgrip was assessed using Spearman's rank correlation coefficient. Unless otherwise stated, all variables are *n* = 16 healthy and *n* = 16 FILD.

Data are expressed as the mean ± standard deviation unless stated otherwise. A value of *P* < 0.05 was considered statistically significant. Statistical analysis was performed using SPSS software, version 27 (IBM, Armonk, NY, USA).

## 3 RESULTS

### 3.1 Study participants

Participant characteristics are presented in Table 1. There were no differences in anthropometric measurements between the healthy and FILD groups. The FILD group had significantly lower lung volumes and a higher dyspnoea rating. The most frequent FILD diagnosis was IPF (*n* = 6). Other diagnoses were connective tissue disease‐ILD (*n* = 5), unclassifiable ILD (*n* = 2), asbestosis (*n* = 1), interstitial pneumonia with autoimmune features (*n* = 1) and cryptogenic organizing pneumonia (*n* = 1). Co‐morbidities in the FILD group were coronary artery disease (*n* = 4), type 2 diabetes (*n* = 2), atrial fibrillation (*n* = 3), structural heart disease (*n* = 3) and hypothyroidism (*n* = 1). Co‐morbidities in the healthy control group were gastroesophageal reflux disease (*n* = 1), anxiety (*n* = 1), Barrett's oesophagus (*n* = 1) and factor V Leiden (*n* = 1). Long‐term prescription medications are presented on Table 2.

**TABLE 1:** Participant characteristics

	Healthy	FILD	*P* value
*N*	16 (four women)	16 (three women)	
Age (year)	67 ± 7	69 ± 14	0.733
Height (cm)	170 ± 10	170 ± 8	0.839
Weight (kg)	73 ± 14	77 ± 9	0.400
BMI (kg/m^2^)	25 ± 3	26 ± 3	0.254
Smoking history (p.y.)	0.7 ± 1	1.2 ± 3	0.572
Resting SBP (mm Hg)	129 ± 11	127 ± 19	0.647
Resting DBP (mm Hg)	79 ± 6	77 ± 8	0.496
Resting MAP (mm Hg)	96 ± 7	94 ± 11	0.540
SpO_2_ (%)	96 ± 1	96 ± 2	0.631
MVC (N)	362 ± 128	304 ± 71	0.121
FEV_1_ (% pred.)	106 ± 11	76 ± 20	0.000
FVC (% pred.)	101 ± 25	71 ± 18	0.001
FEV1/FVC (%)	77 ± 7	82 ± 5	0.012
Dyspnoea in daily living (a.u.)	0.1 ± 0.3	1.2 ± 0.9	0.000

All variables are *n* = 16 healthy and *n* = 16 FILD. Independent sample t‐test used.

Abbreviations: BMI, body mass index; p.y., pack years; SBP, systolic blood pressure; DBP, diastolic blood pressure, MAP, mean arterial pressure, SpO_2_, oxygen saturation; MVC, maximal voluntary contraction; FEV_1_, forced expiratory volume in 1 second; FVC, forced vital capacity; a.u., arbitrary units.

**TABLE 2:** Long‐term prescription medication of participants

Medication	Number of ILD patients	Number of healthy controls
Anti‐fibrotic agents	5	0
Immunosuppressant including prednisone	6	0
ARB or ACEi	5	0
Calcium channel blocker	3	0
Beta‐blocker	3	0
Alpha blocker	2	0
Antiplatelet	6	0
Cholesterol lowering agents	8	2
Anti‐diabetic agents	2	0
Acid suppression therapy	7	1
Anticoagulation	2	1
Other	2^a^	2^b^

All variables are *n* = 16 healthy and *n* = 16 FILD.

Abbreviations: ARB, angiotensin II receptor blocker; ACEi, Angiotensin converting enzyme inhibitor.

^a^ *n* = 1 allopurinol, *n* = 1 thyroxine replacement.

^b^ *n* = 1 anti‐anxiety medication, *n* = 1 anti‐histamine.

### 3.2 Baseline cardiorespiratory variables

As expected, baseline ${\dot{V}}_{E}$ was higher in the FILD group than the healthy group (Table 3). In the FILD group, R*f* was higher whilst $V_{T}$ was similar to the healthy group. P_ET_CO_2_ was lower in the FILD group but SpO_2_ (*n* = 16 healthy, *n* = 15 FILD) was similar between groups. There were no differences in either MAP (*n* = 16 healthy, *n* = 15 FILD) or HR between the healthy and FILD groups. There were no between‐trial differences in any of the measured variables at baseline. Baseline cBRS gain (*n* = 15 healthy, *n* = 11 FILD) and HRV indices (*n* = 16 healthy, *n* = 13 FILD) were not different between the healthy and FILD groups (Table 4). The achieved handgrip force (as a percentage of the individual's MVC) was 55 ± 8% (Control trial) and 55 ± 9% (PECO trial) in the healthy participants, and 54 ± 8% (Control trial) and 54 ± 8% (PECO trial) in the FILD participants.

**TABLE 3:** Baseline cardiorespiratory variables

		Healthy	FILD	ANOVA *P* values		
				Group	Trial	Interaction
${\dot{V}}_{E}$ (L·min^−1^)	Control	11 ± 2	14 ± 3	0.000	0.806	0.738
	PECO	12 ± 2	13 ± 3			
R*f* (breaths·min^−1^)	Control	16 ± 4	19 ± 5	0.000	0.783	0.814
	PECO	15 ± 3	19 ± 4			
V_T_ (L)	Control	0.78 ± 0.27	0.75 ± 0.21	0.275	0.898	0.590
	PECO	0.82 ± 0.23	0.72 ± 0.17			
P_ET_CO_2_ (mm Hg)	Control	43 ± 5	39 ± 3	0.000	0.893	0.735
	PECO	43 ± 5	39 ± 4			
SpO_2_ (%)	Control	96 ± 1	95 ± 2	0.571	0.410	0.778
	PECO	95 ± 1	95 ± 2			
MAP (mm Hg)	Control	97 ± 8	95 ± 11	0.664	0.509	0.688
	PECO	98 ± 8	98 ± 11			
HR (beats·min^−1^)	Control	64 ± 9	68 ± 11	0.123	0.979	0.967
	PECO	64 ± 9	68 ± 12			

All variables are *n* = 16 healthy and *n* = 16 FILD, aside from MAP and SpO_2_ where *n* = 15 FILD. Two‐way analysis of variance used.

Abbreviations: ${\dot{V}}_{E}$, minute ventilation; R*f*, breathing frequency; $V_{T}$, tidal volume; SpO_2_, oxygen saturation; P_ET_CO_2_, end‐tidal carbon dioxide; HR, heart rate; MAP, mean arterial pressure.

**TABLE 4:** Cardiac baroreflex sensitivity and heart rate variability in healthy and FILD participants

	Healthy	FILD	*P* value
***cBRS***			
Gain (ms·mm Hg^−1^)	7.48 ± 3.86	5.11 ± 2.39	0.083
Number of sequences (n)	7 ± 6	4 ± 3	0.113
BEI (%)	0.34 ± 0.20	0.21 ± 0.13	0.080
***HRV***			
RMSSD (ms)	29.8 ± 20.5	30.8 ± 25.2	0.630
SDNN (ms)	26.0 ± 11.5	25.5 ± 17.9	0.486
HF (ms^2^)	310 ± 261	537 ± 833	0.617
LF (ms^2^)	306 ± 356	354 ± 522	0.313
TP (ms^2^)	645 ± 538	931 ± 1116	0.618
HF (n.u.)	55 ± 20	56 ± 23	0.845
LF (n.u.)	45 ± 20	43 ± 23	0.838
LF/HF ratio	1.19 ± 0.88	1.26 ± 1.20	0.806

For cBRS variables *n* = 15 healthy and *n* = 11 FILD. For HRV variables *n* = 16 healthy and *n* = 13 FILD. Independent sample t‐test used.

Abbreviations: BEI, baroreflex effectiveness index; RMSSD, square root of the mean of the sum of successive differences in R–R interval; SDNN, standard deviation of all normal sinus R–R intervals; HF, high frequency; n.u., normalized units; LF, low frequency.

### 3.3 Cardiorespiratory responses

**FIGURE 1:**
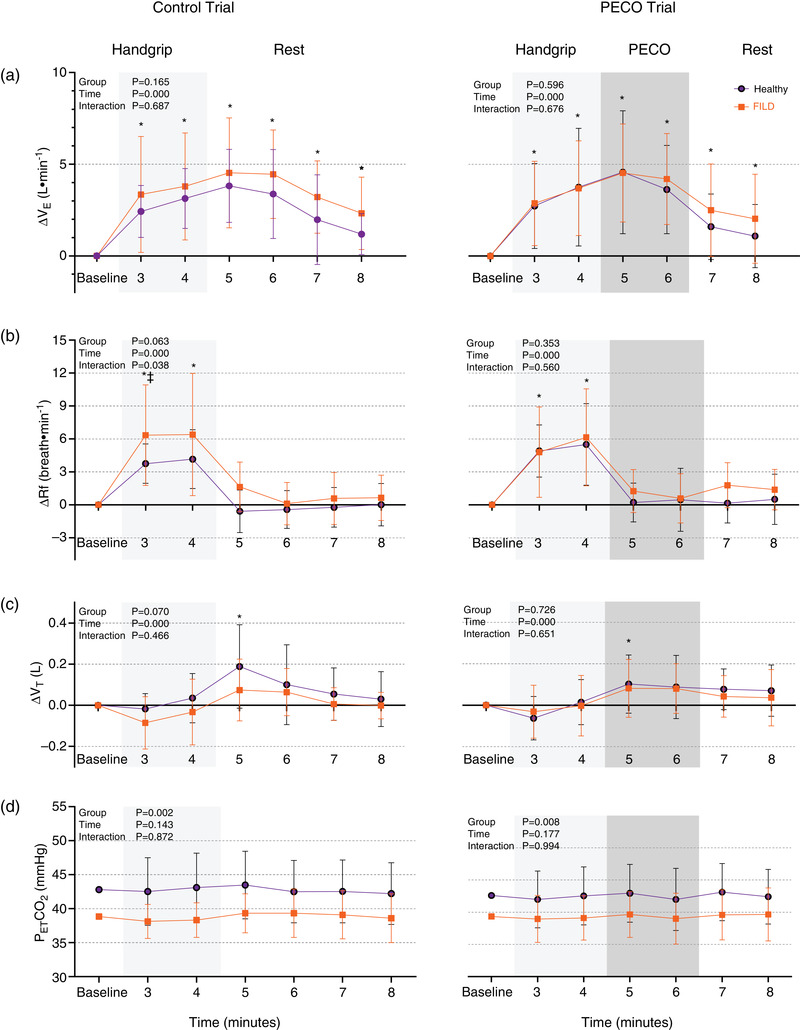
Change in minute ventilation (a; Δ${\dot{V}}_{E}$), respiratory frequency (b; ΔR*f*), tidal volume (c; ΔV_T_) and end‐tidal carbon dioxide (d; P_ET_CO_2_) during Control and PECO trials in healthy and FILD participants. All variables are *n* = 16 healthy and *n* = 16 FILD. Two‐way analysis of variance with repeated measures used. *P < 0.05 versus baseline. ‡ *P *< 0.05 healthy versus FILD groups. Values are means ± standard deviation

${\dot{V}}_{E}$ was increased from baseline during rhythmic handgrip exercise similarly in both the FILD and healthy groups (Figure 1, Table 5). In both groups, this was due to elevated R*f*. In the PECO trial, for example, the mean increase in R*f* in the second min of handgrip was Δ5.5 ± 3.7 breaths·min^−1^ and Δ6.2 ± 4.4 breaths·min^−1^ in the healthy and FILD groups, respectively. Following the cessation of exercise in the Control trial, ${\dot{V}}_{E}$ remained elevated compared to baseline until the second min of recovery. The elevated ${\dot{V}}_{E}$ was predominantly related to elevated V_T_ (*P* = 0.005 compared to baseline). After the second minute of recovery, ${\dot{V}}_{E}$ began to fall but did not return to baseline by the end of the protocol. Similarly, in the PECO trial, ${\dot{V}}_{E}$ continued to increase above baseline in the first min of PECO following the cessation of exercise, driven by elevated V_T_ (*P* = 0.018). ${\dot{V}}_{E}$ began to decline in the second min of PECO but did not reach baseline values by the end of the trial. There were no differences in the ${\dot{V}}_{E}$ responses between the groups (Control trial, *P* = 0.165; PECO trial, *P* = 0.596) or in SpO_2_ (*n* = 16 healthy, *n* = 15 FILD). The P_ET_CO_2_ was higher in the healthy group than the FILD group (*P* = 0.002 in Control and *P* = 0.008 in PECO trial).

**TABLE 5:** Mean respiratory and cardiovascular variables during handgrip exercise, recovery and PECO

		Control		PECO	
		Handgrip	Recovery	Handgrip	PECO
${\dot{V}}_{E}$ (L·min^−1^)	Healthy	15 ± 3	12 ± 2	16 ± 4	13 ± 2
	FILD	18 ± 4	15 ± 3	18 ± 3	15 ± 3
R*f* (breaths·min^−1^)	Healthy	20 ± 4	15 ± 4	21 ± 5	16 ± 5
	FILD	26 ± 4	20 ± 4	25 ± 4	20 ± 5
V_T_ (L)	Healthy	0.81 ± 0.24	0.88 ± 0.39	0.83 ± 0.24	0.90 ± 0.29
	FILD	0.71 ± 0.15	0.81 ± 0.21	0.72 ± 0.15	0.80 ± 0.19
SpO_2_ (%)	Healthy	96 ± 1	97 ± 1	96 ± 1	97 ± 1
	FILD	94 ± 7	94 ± 7	94 ± 7	94 ± 7
SBP (mm Hg)	Healthy	161 ± 25	127 ± 20	162 ± 15	149 ± 14
	FILD	150 ± 26	127 ± 19	153 ± 26	148 ± 21
DBP (mm Hg)	Healthy	96 ± 10	80 ± 8	92 ± 8	87 ± 8
	FILD	86 ± 13	77 ± 10	90 ± 15	87 ± 11
MBP (mm Hg)	Healthy	117 ± 14	96 ± 12	116 ± 10	108 ± 9
	FILD	107 ± 17	94 ± 12	111 ± 18	108 ± 13
PP (mm Hg)	Healthy	65 ± 18	47 ± 14	69 ± 13	63 ± 11
	FILD	60 ± 23	47 ± 19	64 ± 17	61 ± 17
HR (beats·min^−1^)	Healthy	74 ± 14	63 ± 9	74 ± 11	64 ± 10
	FILD	75 ± 13	67 ± 12	74 ± 14	68 ± 12

Handgrip and PECO refer to the last min of the handgrip and PECO periods, respectively. Recovery refers to 6^th^ min of the Control trial (middle of the recovery period). All variables are *n* = 16 healthy and *n* = 16 FILD, aside from SBP, DBP, MAP, Pulse pressure and SpO_2_ where *n* = 15 FILD.

Abbreviations: ${\dot{V}}_{E}$, minute ventilation; R*f*, breathing frequency; $V_{T}$, tidal volume; SpO_2_, oxygen saturation; SBP, systolic blood pressure; DBP, diastolic blood pressure, MAP, mean arterial pressure; PP, pulse pressure; HR, heart rate.

**FIGURE 2:**
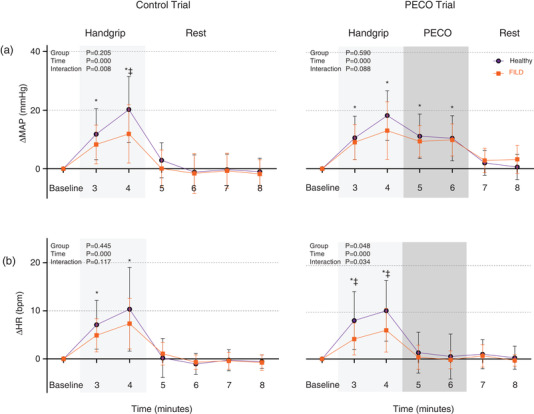
Change in mean arterial blood pressure (a; ΔMAP) and heart rate (b; ΔHR) during Control and PECO trials in healthy and FILD participants. a, *n* = 16 healthy and *n* = 15 FILD; b, *n* = 16 healthy and *n* = 16 FILD. Two‐way analysis of variance with repeated measures used. *P < 0.05 versus baseline. ‡ *P *< 0.05 healthy versus FILD groups. Values are means ± standard deviation

Rhythmic handgrip exercise elevated MAP (*n* = 16 healthy, *n* = 15 FILD) in both FILD and healthy groups (Figure 2, Table 5). In the Control trial, the magnitude of the MAP response was less in the FILD group (11.9 ± 9.9 mm Hg) than in the healthy group (20.2 ± 11.3 mm Hg: *P* = 0.038). After cessation of exercise, MAP returned to baseline in both healthy and FILD groups in the Control trial, whereas MAP remained elevated above baseline levels in the PECO trial. Importantly, the magnitude of the MAP elevation was not different between groups.

Heart rate was also elevated during rhythmic handgrip exercise. In the PECO trial, there was a difference between the groups (group *P* = 0.048, group x time interaction *P* = 0.034) with the magnitude of the HR response to handgrip being less in the FILD group than the healthy group in both the first (Δ4.27 ± 3.41 vs. 8.22 ± 6.20 beats·min^−1^, *P* = 0.033) and second min of handgrip (Δ6.13 ± 4.68 vs. 10.32 ± Δ6.50 beats·min^−1^, *P* = 0.033). When FILD participants with atrial fibrillation/atrial flutter (*n* = 3), in whom chronotropic incompetence was likely, were excluded from analysis, the effect of group was no longer significant (group *P* = 0.075, group x time interaction *P* = 0.810). However, the mean change in HR still tended to be lower in the FILD (first min Δ4.92 ± 3.05 beats·min^−1^, second min Δ5.92 ± 3.86 beats·min^−1^) compared to the healthy group. There was no correlation between cBRS gain and the magnitude of the HR response to handgrip exercise (r_s _= 0.201, *P* = 0.314, *n* = 15 healthy, *n* = 11 FILD), but there was a weak positive correlation between resting RMSSD and the HR response during exercise (r_s _= 0.430, *P* = 0.020). Following cessation of handgrip exercise, HR returned to baseline in both control and PECO trials. The recovery of HR was not different between the groups.

### 3.4 Metaboreflex response

There were no significant differences between the healthy and FILD groups in the ${\dot{V}}_{E}$ (healthy vs. FILD; 0.2 ± 1.6 L·min^−1^ vs. −0.1 ± 1.7 L·min^−1^, *P* = 0.590), MAP (11.4 ± 8.8 mm Hg vs. 11.2 ± 8.1 mm Hg, *P* = 0.961, *n* = 16 healthy, *n* = 15 FILD), and HR metaboreflex responses (1.4 ± 4.7 beats·min^−1^ vs. 0.3 ± 2.6 beats·min^−1^, *P* = 0.425). The metaboreflex contribution to ${\dot{V}}_{E}$ was not significantly correlated with forced vital capacity (r = 0.108, *P* = 0.691), vital capacity (r = −0.046, *P* = 0.864) or diffusing capacity of carbon monoxide (r = 0.019, *P* = 0.944).

### 3.5 Dyspnoea and RPE ratings

The mean dyspnoea rating at baseline was 0.1 ± 0.2 units in the healthy group and 0.3 ± 0.6 units in the FILD group. Handgrip exercise increased the rating in both groups (healthy vs. FILD; Control trial, 0.6 ± 0.8 vs. 1.0 ± 1.1 units, PECO trial, 0.7 ± 0.8 vs. 1.1 ± 1.2 units; all *P* < 0.05 vs. baseline). Following handgrip, in the Control trial the dyspnoea rating declined slightly by the middle of recovery but did not return to baseline ratings (healthy 0.3 ± 0.4 units, FILD 0.8 ± 1.1 units). Similarly, in the PECO trial dyspnoea ratings decreased following cessation of exercise in both groups. At the end of PECO, the dyspnoea rating in the healthy group was 0.5 ± 1.0 units. In comparison, the decrease in the FILD group was minimal (from 1.0 ± 1.2 units to 1.0 ± 1.3 units). Overall, there were no differences in ratings between the groups in the PECO trial (*P* = 0.210). The mean RPE at the end of handgrip exercise was 2.97 ± 3.48 units and 2.14 ± 1.59 units for healthy and FILD groups, respectively (*P* = 0.271).

## 4 DISCUSSION

The aim of this study was to determine whether the cardiorespiratory response to skeletal muscle metaboreflex, achieved with PECO following rhythmic handgrip exercise, was augmented in FILD. The major novel findings are (1) ${\dot{V}}_{E}$ responses to rhythmic handgrip were not different in the FILD and control groups, and while ${\dot{V}}_{E}$ remained elevated during PECO in both groups, there was no difference in the magnitude of this response, (2) dyspnoea ratings to handgrip and PECO were similar between groups, (3) blood pressure responses to handgrip were lower in FILD, but were similarly elevated during PECO, and (4) HR responses to handgrip were lower in FILD and weakly associated with resting indices of cardiac parasympathetic activity (i.e., RMSSD). Collectively, these findings suggest that the skeletal muscle metaboreflex contribution to the respiratory and cardiovascular response to exercise is not altered in FILD.

### 4.1 Ventilation and dyspnoea

Several mechanisms have been hypothesised to contribute to exercise hyperpnoea (Forster et al., 2011). A number of earlier studies of isolated metaboreflex activation in healthy individuals did not observe a maintenance of the exercise‐induced elevation in ${\dot{V}}_{E}$ during PECO (Fukuba et al., 2007; Haouzi et al., 1993; Rowell et al., 1976), supporting the view that afferent feedback from contracting muscle was not an important mechanism of exercise hyperpnoea (Waldrop et al., 2010). However, recent research has challenged this assumption. First, non‐selective inhibition of skeletal muscle afferent feedback during exercise by lumbar intrathecal fentanyl (μ‐opioid receptor agonist) resulted in hypopnea and elevated P_ET_CO_2_. Taking into account the stimulatory effects of hypercapnia on ventilation, such afferent blockade was able to reduce exercise hyperpnoea by 15–49% (Amann et al., 2010). More specific evidence for the metaboreflex being a driver of exercise hyperpnoea was provided by (Lam et al., 2019). In this protocol left‐legged cycling exercise was followed by 3 min of either PECO or free‐flow recovery of the leg, while right leg cycling was performed. ${\dot{V}}_{E}$ relative to baseline was higher in the PECO compared to free‐flow recovery condition. Furthermore, PECO following handgrip and calf plantar‐flexion exercise was shown to stimulate ventilation under conditions of concurrent mild hypercapnia (Bruce & White, 2012; Lykidis et al., 2010). Collectively, these studies support the involvement of the skeletal muscle metaboreflex in the regulation of exercise hyperpnoea. However, the presence of other stimulatory inputs appear to be a prerequisite, which suggest a synergistic interaction between the metaboreflex and other respiratory inputs (White & Bruce, 2020).

Isolated metaboreflex using PECO in some chronic disease states, such as COPD and heart failure, results in elevated ${\dot{V}}_{E}$ (Bruce et al., 2016; Piepoli et al., 1996), suggesting a link between augmented metaboreflex sensitivity and heightened exercise hyperpnoea in these patients. However, contrary to our hypothesis, we did not find evidence that metaboreflex activation results in elevated ventilation in FILD compared to healthy controls. When interpreting our data, it is important to exclude concurrent influences on respiratory drive and limitations in the respiratory system's ability to increase ventilation. Neither group developed hypoxia at any stage during the trials and there was no difference in SpO_2_ between the groups. The P_ET_CO_2_, used as a surrogate of the arterial partial pressure of CO_2_, was lower in the FILD group at baseline; however, this remained stable during the exercise and PECO periods, making increased central and/or peripheral chemoreflex activation unlikely. We acknowledge that the P_ET_CO_2_ may not provide as accurate estimate of arterial CO_2_ with increasing age (Satoh et al., 2015) and ILD (Enomoto et al., 2021). During PECO, both groups maintained an elevated MAP of ∼10 mm Hg suggesting that the level of metaboreflex activation was similar in both groups. Furthermore, this magnitude of pressor response is in line with other studies that used a similar protocol (Bruce et al., 2016; Sherman et al., 2011), hence confirming the validity of our protocol. Finally, even though FILD decreases the respiratory system's capacity to increase ventilation (Faisal et al., 2016), considering the relatively low absolute maximum ${\dot{V}}_{E}$ attained we conclude that mechanical restriction was unlikely to contribute to the observed ventilatory response.

Our results in FILD can be compared with findings in COPD, a respiratory disease also associated with dyspnoea and exercise intolerance. The study by Bruce et al. (2016) in moderate‐to‐severe COPD used the same protocol which allows for direct comparisons. In both studies, rhythmic handgrip exercise produced rapid increase in ${\dot{V}}_{E}$ in all participants. However, the magnitude of the ventilatory response was substantially greater in the Bruce et al. (2016) study. For example, the maximum Δ${\dot{V}}_{E}$ with exercise was ∼6 and ∼8 L·min^−1^ in the control and COPD groups, which is approximately twice that seen in our control and FILD participants. Contrary to our findings in FILD, in COPD patients the ${\dot{V}}_{E}$ remained significantly above baseline throughout the PECO period. This finding was present in both hypercapnic and normocapnic patients, supporting the concept of abnormal involvement of the metaboreflex in ventilatory control in COPD. However, a more recent study using PECO following static handgrip exercise in COPD failed to find an augmented ventilatory response (Iepsen et al., 2021). Possible explanations for the result are the small sample size of this study (respiratory data available in *n* = 8) and the use of a pneumobelt which only provides indirect measurement of respiratory variables.

Comparisons in the pattern of the ventilatory response warrant further discussion. Firstly, in our study the ${\dot{V}}_{E}$ remained elevated in the free‐flow recovery period immediately following exercise. This is in contrast to the pattern seen in Bruce et al. (2016) and other studies of similar methodology (Lykidis et al., 2010; Piepoli et al., 1996), where ${\dot{V}}_{E}$ declined rapidly following cessation of exercise (although still remained above baseline). This discrepancy may be attributable to the relative smaller increase in ${\dot{V}}_{E}$ during handgrip exercise in the present study. Alternatively, excessive participant attention or a degree of entrainment of the respiratory rate to the handgrip rhythm may contribute; however, neither explanation seems likely as participants were carefully familiarised and there were no significant fluctuations in the P_ET_CO_2._ Nevertheless, in the PECO trial the ventilatory response were similar between the groups, therefore our conclusion remains unchanged. Secondly, an intriguing finding in both our study and Bruce et al. (2016) was the differential ventilatory responses to exercise and PECO/rest. During exercise the rise in ${\dot{V}}_{E}$ was primarily due to elevated R*f* and during PECO/rest the rise in ${\dot{V}}_{E}$ was predominantly secondary to elevated V_T._ This is supportive of the view of differential neural control of the R*f* and V_T_ (Nicolò et al., 2018; Tipton et al., 2017).

Dyspnoea is defined as ‘a subjective experience of breathing discomfort that consists of qualitatively distinct sensations that vary in intensity’ (Parshall et al., 2012). Despite being a subjective sensation, it is widely accepted that exertional dyspnoea is related to exercise hyperpnoea (Parshall et al., 2012), as most humans will report dyspnoea at some point as exercise intensity and ${\dot{V}}_{E}$ increases (Dempsey et al., 2010). Dyspnoea is consciously perceived when dyspnoeic signals from the medullary respiratory centres project to sensory regions in the cerebral cortex (Banzett et al., 2021). Skeletal muscle afferents, which stimulate ventilation, have been hypothesized to contribute to dyspnoea either through parallel projections to the sensory cortex, or through an integrated signal that ascend from the brainstem (termed ‘corollary discharge’) (Banzett et al., 2021; Forster et al., 2011). Indeed, the attenuation of lower limb muscle afferents with intrathecal fentanyl in COPD reduced dyspnoea during cycling exercise (Gagnon et al., 2012). Dyspnoea was not assessed in previous studies employing handgrip and PECO in COPD (Bruce et al., 2016; Iepsen et al., 2021). We did not find differences in dyspnoea ratings (0–10 Borg scale) between the FILD and control groups during isolated metaboreflex activation. The increase in dyspnoea rating achieved from rhythmic handgrip exercise was small (∼0.5–1 unit), and therefore it is possible that a difference could be revealed if a higher handgrip intensity or a different exercise modality were used. Although the Borg Category Ratio Scale is a widely accepted instrument to quantify dyspnoea (Society AT & Physicians ACoC, 2003), dyspnoea is increasingly recognised to comprise of distinct sensations (Banzett et al., 2021; Parshall et al., 2012) which is more effectively assessed with multidimensional assessment tools (e.g., Banzett et al., 2015).

### 4.2 Blood pressure

All participants demonstrated a substantial blood pressure response to handgrip exercise, a finding that is consistent with established literature (Delius et al., 1972; Lind et al., 1964). The cardiovascular response to exercise reflects the interaction between descending motor outflows from higher brain centres (central command), reflexes from skeletal muscle and the arterial baroreflex (Kaufman & Forster, 1996). Blood pressure is increased through the modulation of autonomic neural flow to the heart and peripheral vasculature (Smith et al., 2006). In the Control trial, the data suggests that the pressor response to exercise is attenuated in FILD compared to healthy controls. The reported RPE was similar between groups, suggesting that central command was similar. There was unlikely to be significant differences in the contribution of the metaboreflex because during the PECO period, when only the metaboreflex was active, the MAP response was almost identical between the groups. A possible explanation for the differential pressor response is that muscle mechanoreceptors are desensitised in FILD. In addition, evaluation of muscle sympathetic nerve activity could also be helpful to explore the mechanisms involved.

### 4.3 Heart rate

Heart rate increases during exercise are crucial to sustaining exercise (Brubaker & Kitzman, 2011) and an inability to increase HR, or chronotropic incompetence, is associated with exercise intolerance and mortality in both healthy (Lauer et al., 1996; Sandvik et al., 1995) and ILD populations (Holland et al., 2013; Miki et al., 2003; Swigris et al., 2011). Despite this, limited studies have evaluated HR responses to exercise in ILD. These studies demonstrate that during cardiopulmonary exercise testing, patients tend to have a variable HR response with higher HR for a given exercise intensity but below age predicted values at peak exercise (Molgat‐Seon et al., 2019). We observed a reduced HR response in the FILD group which was significant in the PECO trial. We considered whether this could be attributed to co‐morbidities. Atrial fibrillation is associated with chronotropic incompetence and the use of heart rate‐limiting medication. However, when these participants were removed from the analysis the trend for a reduced HR response to exercise remained. Another consideration is resting cardiac vagal tone. Cardiac vagal outflow is the major determinant of the HR response to exercise, as tachycardia is mediated, in part, by the withdrawal of cardiac parasympathetic outflow exercise (Fisher et al., 2015). A reduction in the HR response to isometric exercise is seen with normal aging (Goldstraw & Warren, 1985; Kaijser & Sachs, 1985; Taylor et al., 1991) and this has been associated with a decrease in resting cardiac vagal tone (Hellman & Stacy, 1976; Korkushko et al., 1991). Neither baseline HRV nor cBRS gain, indices of cardiovagal activity, were different in FILD and age‐matched healthy controls. However, the HR response to handgrip was positively correlated with RMSSD, supporting the concept that resting cardiac vagal tone influences the HR response to exercise and may contribute to the between group differences observed.

In contrast to the robust blood pressure response generated from metaboreflex activation during PECO, HR returned toward baseline levels in both groups. This is consistent with other studies of PECO following rhythmic or isometric handgrip exercise (Fisher et al., 2010; Mark et al., 1985; Nishiyasu et al., 1994) and is likely attributable to the elevated blood pressure during PECO activating the arterial baroreflex and increasing cardiac parasympathetic activity (Fisher et al., 2010; O'Leary, 1993).

### 4.4 Methodological considerations

We used rhythmic handgrip exercise to activate the skeletal muscle metaboreflex. This paradigm is well established and accessible for FILD patient who are often frail, and we considered that it would, in contrast to isometric exercise, help patients avoid inadvertent straining manoeuvres and volitional changes to their breathing pattern. On the other hand, the magnitude of the cardiorespiratory response to exercise varies with the exercise modality and the muscle mass engaged (Fisher et al., 2015; Forster et al., 2011; Freund et al., 1978). Therefore, a protocol that engaged a larger muscle mass (e.g., leg cycling) would be expected to generate greater ventilatory responses. We did not use whole‐body exercise as the ventilation achieved is likely to be affected by mechanical constraints as a result of pathological reduction in maximum voluntary ventilation in FILD (Faisal et al., 2016). We note that the perceived effort and dyspnoea with the handgrip paradigm used were low. As participants were asked to provide a rating immediately after (but not during) the trial this may be a potential limitation. However, this approach was considered superior to assessing these values during the protocol while participants were instrumented with a full‐face mask and when non‐verbal methods of assessment could lead to inadvertent increase in subject awareness of the assessed variables.

It should also be noted that the FILD population in our study had several co‐morbidities and were taking prescription medications. These factors cannot be ruled out as potentially influencing the results of our study. Ideally, the groups should be matched by co‐morbidities and medications. However, this is not feasible due to the relative rarity of FILD and high prevalence of co‐morbidities in FILD patients (Collard et al., 2012; Raghu et al., 2015). Our approach of selecting a control group with minimal co‐morbidities is consistent with other ILD physiology studies (Milne et al., 2020; Schaeffer et al., 2018). In addition, studies on the effect of cardiometabolic diseases on metaboreflex activation have shown exaggerated responses (Amann et al., 2014; Chant et al., 2018; Holwerda et al., 2016; Piepoli et al., 1996), in many instances even when chronic medications are continued. As our study did not demonstrate an altered metaboreflex activation in FILD, this does not support the interpretation that co‐morbidities and/or medications had significant effect on the primary outcomes.

### 4.5 Perspectives

Exertional dyspnoea and exercise intolerance have a profound impact on those living with ILD (Collard & Pantilat, 2008; De Vries & Drent, 2006). Despite this, management options are limited and frequently inadequate. Treatments that have been effective in other respiratory conditions have been less successful in ILD. Pulmonary rehabilitation, a cornerstone in the management of COPD (McCarthy et al., 2015) produced only modest, short‐lived improvement in exercise tolerance in ILD (Holland et al., 2008; Kozu et al., 2011). The effect of supportive therapies, such as ambulatory oxygen and systemic opioids in ILD has produced mixed and disappointing results (Allen et al., 2005; Kronborg‐White et al., 2020; Sharp et al., 2016). Moreover, these options are associated with limitations such as psychosocial stigma and medication side effects (Khor et al., 2017; Kronborg‐White et al., 2020). These highlight the need for a deeper understanding of the mechanisms of exercise limitation in ILD and the defining of novel therapeutic targets. Our study contributes to this by demonstrating that the skeletal muscle metaboreflex is unlikely to have a strong contribution to exercise limitation in FILD. The role of other sensory afferents should be explored in future studies.

## COMPETING INTERESTS

The authors have no conflicts of interest to disclose.

## AUTHOR CONTRIBUTIONS

Experiments were performed in the Human Cardiorespiratory Physiology Laboratory, Department of Respiratory Physiology, Auckland City Hospital, Auckland District Health Board. Charlotte Chen, John Kolbe, Julian F. R. Paton and James P. Fisher contributed to conception or design of the work. All authors contributed to the acquisition, analysis or interpretation of data for the work and drafting the work or revising it critically for important intellectual content. All authors approved the final version of the manuscript and agree to be accountable for all aspects of the work. All persons designated as authors qualify for authorship, and all those who qualify for authorship are listed.

## Supporting information

Statistical Summary Document

## Data Availability

Data is available upon reasonable request to the corresponding author.
