# Supplementary material for: Cardiorespiratory responses to muscle metaboreflex activation in fibrosing interstitial lung disease
Source: Exp Physiol. 2022 Mar 30;107(5):527–40. doi: 10.1113/EP090252 (PMC9314965; doi:10.1113/EP090252)
Supplement: Supplementary file 1 — Statistical Summary Document [file EPH-107-527-s001.docx]

**Manuscript Title:** Cardiorespiratory responses to muscle metaboreflex activation in fibrosing interstitial lung disease

**Authors:** Charlotte Chen; John Kolbe; Margaret L Wilsher, Sally De Boer, Julian F R Paton; James P Fisher

**Animal model used, if applicable:**

**Underlying hypothesis:** This investigation tests the hypothesis isolated metaboreflex activation in fibrosing interstitial lung disease (FILD) would result in elevated ventilation (V̇_E_) and dyspnoea ratings compared to healthy controls, due to augmented muscle metaboreflex.

**Definitions of ‘n’:** Total subjects n=32, FILD n=16, healthy controls n=16

**Statistical summary table:**

| Experimental question number* | Finding/ conclusion | Experimental location/ variable  e.g. muscle, neocortex or genotype | Mean value  (or other summary statistic) | SD | n val. | P** | Units | Data comparisons  e.g. WT vs KO | Statistical test | Any other variable  e.g. subjects’ age or sex | Figure/ table in which data are presented | Comments  e.g. observation |
| --- | --- | --- | --- | --- | --- | --- | --- | --- | --- | --- | --- | --- |
| 1. Did V̇_E_ differ between FILD than healthy control? | Similar magnitude of change V̇_E_ in both groups | Control trial | Mean ΔV̇_E_, healthy vs. FILD  Baseline0±0vs 0 ±0  3min:2.4±1.4vs 3.3:±3.2  4min:3.1±1.6vs 3.8±2.9  5min:3.8±2.0vs 4.5±3.0  6min:3.4±2.4vs 4.5±2.4  7min:2.0±2.5vs 3.2±2.0  8min:1.2±1.1vs 2.3±2.0 | Listed in previous column | healthy=16  FILD n=16 | Group P=0.165  Time P=0.000  Interaction P=0.687 | L·min^-1^ | Healthy vs FILD  Time vs baseline | 2 way ANOVA with repeated measures |  | Fig 1, table 5 |  |
|  |  | PECO trial | Baseline0±0vs 0 ±0  3min:2.7±2.3vs 2.9±2.3  4min:3.8±3.2vs 3.7±2.59  5min:4.6±3.4vs 4.5±2.67  6min:3.6±2.4vs 4.2±2.48  7min:1.6±1.8vs 2.5±2.52  8min:1.1±1.7vs 2.0±2.43 |  | healthy n=16  FILD n=16 | Group P=0.596  Time P=0.000  Interaction P=0.676 | L·min^-1^ | Healthy vs FILD  Time vs baseline |  |  |  |  |
| 2 Did oxygen saturation (SpO_2_) differ between FILD and healthy control? | No between group difference | Control trial | healthy vs. FILD  Baseline 96±1vs 96±2  3min 96±1vs 95 ±2  4min 96±1vs 95 ±3  5min 97±1vs 96 ±3  6min 97±1vs 96 ±3  7min 96±1vs 96 ±2  8min 96±1vs 96 ±2 | Listed in previous column | Healthy n=16  FILD n=15 | Group P=0.278  Time P=0.000  Interaction P=0.00528 | % | Healthy vs FILD,  Time vs baseline | 2 way ANOVA with repeated measures | Post hoc pairwise comparison (Bonferroni correction)  All P>0.05 | Within text, table 5 |  |
|  |  | PECO trial | Baseline95±1vs 95±3  3min 96±1vs 95±3  4min 96±1vs 96±3  5min 97±1vs 95±3  6min 97±1vs 96±3  7min 97±1vs 95±3  8min 96±1vs 96±3 |  |  | Group P=0.262  Time P=0.000  Interaction P=0.0400 |  |  |  | Post hoc pairwise comparison (Bonferroni correction)  All P>0.05 |  |  |
| 3. Did respiratory frequency (R*f*) differ between FILD than healthy control? | FILD R*f* was higher than healthy controls in the 3^rd^ min of the control trial. No difference in PECO trial | Control trial | Mean ΔR*f*, healthy vs. FILD  Baseline0.0±0.0 vs 0.0±0.0  3min:3.8±1.8vs 6.4±4.6  4min:4.2±2.7vs 6.4±5.6  5min: -0.6±1.9vs 1.6±2.3  6min:-0.4±1.7vs 0.1±1.9  7min:-0.2±1.8 vs 0.6±2.4  8min:0.0±1.9vs 0.6±2.1 | Listed in previous column | healthy n=16  FILD n=16 | Group P=0.0627  Time P=0.000  Interaction P=0.0385 | breaths·min^-1^ | Healthy vs FILD  Time vs baseline | 2 way ANOVA with repeated measures |  | Fig 1, table 5 |  |
|  |  | PECO trial | Baseline0.0±0.0 vs 0.0±0.0  3min:4.9±2.4vs 4.8±4.1  4min:5.5±3.7vs 6.2±4.4  5min:0.2±1.8vs 1.3±2.0  6min:0.5±2.9vs 0.6±2.3  7min:0.2±1.8vs 1.8±2.1  8min:0.5±2.3vs 1.4±1.8 |  |  | Group P=0.353  Time P=0.000  Interaction P=0.560 |  |  |  |  |  |  |
| 4. Did tidal volume (V_T_)differ between FILD and healthy control? | No between group difference | Control trial | Mean Δ V_T_, healthy vs. FILD  Baseline0.00±0.00vs 0.00±0.00  3min:-0.02±0.07vs -0.09±0.13  4min:0.03±0.12 vs -0.03±0.16  5min:0.19±0.20 vs 0.07±0.15  6min:0.10±0.19vs 0.06±0.11  7min:0.05±0.13vs 0.01±0.08  8min:0.03±0.13 vs 0.00±0.06 | Listed in previous column | healthy n=16  FILD n=16 | Group P=0.0704  Time P=0.000  Interaction P=0.466 | L | Healthy vs FILD  Time vs baseline | 2 way ANOVA with repeated measures |  | Fig 1, table 5 |  |
|  |  | PECO trial | Baseline0.00±0.00vs 0.00±0.00  3min:-0.06±0.10vs -0.03±0.13  4min:0.01±0.11vs 0.00±0.15  5min:0.10±0.14 vs 0.08±0.14  6min:0.09±0.15 vs 0.08±0.12  7min:0.08±0.10vs 0.04±0.10  8min:0.07±0.12 vs 0.04±0.14 |  |  | Group P=0.726  Time P=0.000  Interaction P=0.651 |  |  |  |  |  |  |
| 5. Did end tidal CO2 (P_ETCO2_) differ between FILD and healthy control? | P_ETCO2_ is higher in healthy controls | Control trial | Mean P_ETCO2_, healthy vs.FILD  Baseline:43vs 39  3min:42±5vs 38 ±3  4min:43±5vs 38 ±3  5min:43±5vs 39 ±3  6min:42±5vs 39 ±4  7min:42±5vs 39 ±4  8min:42±5vs 38 ±4 | Listed in previous column | healthy n=16  FILD n=16 | Group P=0.00189  Time P=0.143  Interaction P=0.872 | mmHg | Healthy vs FILD  Time vs baseline | 2 way ANOVA with repeated measures |  | Fig 1, table 5 |  |
|  |  | PECO trial | Baseline:43vs 39  3min:42±4vs38 ±4  4min:42±5vs 39 ±3  5min:43±4vs 39 ±4  6min:42±5vs 39 ±4  7min:43±4vs 39 ±4  8min:42±4vs 39 ±4 |  |  | Group P=0.00768  Time P=0.177  Interaction P=0.994 |  |  |  |  |  |  |
| 6. Did mean arterial pressure (MAP) differ between FILD and control? | In the control trial, Δ MAP was higher in the healthy controls in the 4^th^ min, compared to FILD. Δ MAP was similar between groups in the PECO trial. | Control trial | Mean Δ MAP, healthy vs. FILD  Baseline:0.0±0.0vs 0.0±0.0  3min:11.8±8.7vs 8.3±6.6  4min:20.2±11.3 vs 11.9±9.9  5min:2.9±5.9vs 0.1±6.3  6min:-1.2±5.9vs -1.6±6.8  7min:-0.3±5.3vs -0.7±6.0  8min:-1.0±4.6vs -1.8±4.9 | Listed in previous column | Healthy n=16  FILD n=15 | Group P=0.205  Time P=0.000  Interaction P=0.00763 | mmHg | Healthy vs FILD  Time vs baseline | 2 way ANOVA with repeated measures |  | Fig 2, table 5 |  |
|  |  | PECO trial | Baseline:0.0±0.0vs 0.0±0.0  3min:10.6±7.5vs 9.1±6.0  4min:18.3±8.5vs 13.1±9.9  5min:11.2±7.6vs 9.4±5.4  6min:10.5±7.7vs 9.9±5.5  7min:2.0±4.3vs 2.9±4.2  8min:0.6±4.3vs 3.2±4.8 |  |  | Group P=0.590  Time P=0.000  Interaction P=0.0880 |  |  |  |  |  |  |
| 7. Did heart rate (HR) differ between FILD than healthy control? | In the control trial, Δ HR was similar between groups. In the PECO trial, Δ HR was higher in the 3^rd^ and 4^th^ min in the healthy controls compared to FILD. | Control trial | Mean Δ HR, healthy vs. FILD  Baseline: 0.0±0.0 vs0.0±0.0  3min:7.1±5.1 vs 4.9±3.5  4min:10.3±8.7 vs7.3±5.3  5min:0.2±4.1 vs1.1±2.4  6min:-1.1±2.2 vs-0.7±1.5  7min:-0.3±2.2 vs-0.4±1.8  8min:-0.6±1.4 vs-0.8±1.6 | Listed in previous column | Healthy n=16  FILD n=16 | Group P=0.445  Time P=0.000  Interaction P=0.117 | beats·min^-1^ | Healthy vs FILD  Time vs baseline | 2 way ANOVA with repeated measures |  | Fig 2, table 5 |  |
|  |  | PECO trial | Baseline:0.0±0.0 vs0.0±0.0  3min:8.2±6.2 vs4.3±3.4  4min:10.3±6.5 vs6.1±4.7  5min:1.3±4.4vs 0.4±2.6  6min:0.5±4.8vs -0.2±1.9  7min:1.0±3.1 vs0.7±2.4  8min:0.3±2.5 vs-0.3±1.2 |  |  | Group P=0.0483  Time P=0.000  Interaction P=0.0345 |  |  |  |  |  |  |
| 8. Did baseline cardiac baroreflex sensitivity differ between FILD than healthy control? | No difference between groups |  | Healthy vs FILD  Gain 7.48 ± 3.86vs5.11 ± 2.39  BEI 0.34 ± 0.20vs0.21 ± 0.13 | Listed in previous column | Healthy n=16  FILD n=12 | Gain: P=0.0829  BEI P=0.0802 | ms·mmHg-1  (%) | Healthy vs FILD | Unpaired T-test |  | Table 4 |  |
| 9. Did baseline heart rate variability differ between FILD than healthy control? | No difference between groups |  | Healthy vs FILD  RMSSD 29.8 ± 20.530.8 ± 25.2  SDNN 26.0 ± 11.525.5 ± 17.9 | Listed in previous column | Healthy n=16  FILD n=13 | RMSSD P=0.630  SDNN P=0.486 | ms | Healthy vs FILD | RMSSD-Mann-Whitney U test  SDNN- unpaired t- test |  | Table 4 |  |
| 12. Was there a difference in metaboreflex response between FILD and healthy control? | No difference in metaboreflex response between groups | Ventilation | Healthy vs FILD, 0.2 ± 1.6vs-0.12 ± 1.7 | Listed in previous column | Healthy n=16, FILD n=16 | 0.590 | L·min^-1^ | Healthy vs FILD | Unpaired t-test |  | In text |  |
|  |  | Mean arterial pressure | Healthy vs FILD, 11.4 ± 8.8vs 11.2 ± 8.1 | Listed in previous column | Healthy n=16, FILD n=15 | 0.961 | mmHg |  |  |  |  |  |
|  |  | Heart rate | Healthy vs FILD, 1.4 ± 4.7vs 0.3 ± 2.6 | Listed in previous column | Healthy n=16, FILD n=16 | 0.425 | beats·min^-1^ |  |  |  |  |  |
| 14. Did overall dyspnoea ratings differ between FILD and healthy control? | No between group difference | Control trial | Healthy vs FILD  Baseline: 0.1±0.2vs0.3±0.6  End of exercise: 0.6±0.8vs1.0±1.1  Mid-recovery 0.3±0.4vs0.8±1.1 | Listed in previous column | Healthy n=16, FILD n=16 | Group P=0.139  Time P=0.000  Interaction P=0.444 | Borg units | Healthy vs FILD, time vs baseline | 2 way ANOVA with repeated measures |  | In text |  |
|  |  | PECO trial | Baseline: 0.1±0.2vs0.3±0.6  End of exercise: 0.7±0.8vs1.1±1.2  End of PECO:0.5±1.1vs1.0±1.3 |  |  | Group P=0.210  Time P=0.000  Interaction P=0.569 |  |  |  |  |  |  |
| 15. Did overall rated perceived exertion ratings differ between FILD and healthy control? | No between group difference |  | Healthy vs FILD  2.97 ± 3.48 vs 2.14 ± 1.59 | Listed in previous column | Healthy n=16, FILD n=16 | 0.271 | Borg units | Healthy vs FILD, | Unpaired T test |  | In text |  |

*You may use multiple lines for the same question to indicate multiple comparisons

** Authors may wish to make the text bold where p is considered significant against a stated confidence limit.
